# Unfolding similarity in interphysician networks: the impact of institutional and professional homophily

**DOI:** 10.1186/s12913-015-0748-9

**Published:** 2015-03-10

**Authors:** Daniele Mascia, Fausto Di Vincenzo, Valentina Iacopino, Maria Pia Fantini, Americo Cicchetti

**Affiliations:** Department of Public Health and ALTEMS (Graduate School of Health Economics and Management), Catholic University of the Sacred Heart, Largo F. Vito 1, 00168 Rome, Italy; Department of Economic Studies, G. d’Annunzio University, Viale Pindaro 42, 65127 Pescara, Italy; Department of Economics and Management and ALTEMS (Graduate School of Health Economics and Management), Catholic University of the Sacred Heart, Largo F. Vito 1, 00168 Rome, Italy; Department of Public Health, University of Bologna, Via San Giacomo 12, 40126 Bologna, Italy

**Keywords:** Homophily, Physicians’ networks, Social network analysis, Organizational theory

## Abstract

**Background:**

Modern healthcare is characterized by high complexity due to the proliferation of specialties, professional roles, and priorities within organizations. To perform clinical interventions, knowledge distributed across units, directorates and individuals needs to be integrated. Formal and/or informal mechanisms may be used to coordinate knowledge and tasks within organizations. Although the literature has recently considered the role of physicians’ professional networks in the diffusion of knowledge, several concerns remain about the mechanisms through which these networks emerge within healthcare organizations. The aim of the present paper is to explore the impact of institutional and professional homophilies on the formation of interphysician professional networks.

**Methods:**

We collected data on a community of around 300 physicians working at a local health authority within the Italian National Health Service. We employed multiple regression quadratic assignment procedures to explore the extent to which institutional and professional homophilies influence the formation of interphysician networks.

**Results:**

We found that both institutional and professional homophilies matter in explaining interphysician networks. Physicians who had similar fields of interest or belonged to the same organizational structure were more likely to establish professional relationships. In addition, professional homophily was more relevant than institutional affiliation in explaining collaborative ties.

**Conclusions:**

Our findings have organizational implications and provide useful information for managers who are responsible for undertaking organizational restructuring. Healthcare executives and administrators may want to consider the structure of advice networks while adopting new organizational structures.

## Background

Coordination and work in healthcare increasingly occur through informal networks of relationships rather than through channels that are tightly prescribed by formal reporting structures or detailed work processes [1]. Physicians often establish interpersonal collaborative ties with colleagues to access and exchange clinical knowledge and to solve daily problems or decide on effective treatments for patients [2,3]. The healthcare management literature has widely investigated the relevance of social networks within communities of professionals [4-6]. The preponderance of prior research focuses on the structure and impact of social networks on individual and organizational outcomes and benefits, such as quality of care, adoption of innovations and evidence-based medicine (EBM), patient satisfaction, and prescribing behavior [7-12]. Social network analysis (SNA) has been used to explain information exchange patterns among professionals, such as the diffusion of medical innovations [3,13,14], physicians’ decision making [15] as well as organizational culture and hierarchies [2,16]. Despite the wide application of SNA in healthcare, less attention has been paid to the determinants of social network formation among physicians [17,18].

The propensity to create professional relationships is strongly driven by homophily that is, the preference of individuals to choose others who are similar to themselves as partners [19]. Increasing interest in this topic is due to the growing relevance of interactions among individuals in sociological and organizational issues [20]. Different attributes have been identified as determinants of homophily, including race and ethnicity, gender, age, religion, education, occupation and social class, network positions, behaviors, attitudes, abilities, beliefs, and aspirations [19]. Moreover, homophily has been studied in several settings, such as voluntary organizations [21], postgraduate educational programs, universities and schools [22-25], hospitals [26], workplace organizations [27] and courthouses [28].

The principle that “similarity breeds connection” [19] simplifies the process of communication, by mitigating conflicts and relationship costs and by producing relevant effects in terms of trust and solidarity [20,24,25]. Homophily usually arises from an individual’s choice (“choice homophily”) or from the specific structure of the individual’s social world (“induced homophily”) [21]. In the first case, the selection occurs because of the preference of similar attributes; in the second case, relational choices are guided by the social context. In other words, the creation of homophilous ties can be due to individual preference as well as to social world proposals, even if the latter are the results of choices and opportunities along an individual’s life [24].

In the healthcare sector, professional categories delineate the boundaries of the social space in which collective norms, rules and behaviors are generated and adopted by individuals [5]. For example, belonging to the same medical specialty largely explains the propensity of physicians to form professional networks in hospital organizations [29] and to ensure fruitful peer-to-peer communication [30]. On the other hand, physicians are often enrolled in separate clusters with highly similar members. Thus, professional boundaries may constitute a problem when they hinder the spread of innovations, guidelines, and clinical protocols [5]. The social environment—that is, the physical and institutional spaces where individuals interact—is a relevant issue affecting homophilous ties, which can induce similarity in behaviors [8]. In the healthcare sector, such an institutional space could refer to emerging organizational models characterizing hospital organizations.

Many Western healthcare systems (e.g., U.K., Italy, Australia, France) have implemented healthcare reforms aimed at the adoption of new organizational models that focus on fostering patient-centered care and a team-based approach in the development of clinical activities [31,32]. These newly adopted models, referred to as “clinical directorates” or “departments”, are defined by groups of clinical specialties that are integrated with the specific purpose of changing the routine behaviors of professionals within hospitals. For example, clinical directorates were introduced into the Italian National Health System (I-NHS) as an institutional reference model, with the aim of reorienting activities towards healthcare processes carried out by divisional units [33] in charge of making strategic and organizational decisions [34,35]. These intermediate organizational models manage certain services of large hospitals and resemble the divisions that are typically adopted in large private multinational corporations [31,32]. The size of the directorate, its degree of autonomy, and the criteria used for integrating hospital clinical wards may vary considerably across health systems [32]. Hospital executives are often free to decide how to implement the new model in hospitals by selecting the specific clinical wards and medical specialties to be integrated into single departments. The variety of merged clinical specialties heavily affects the ability of clinical directorates to influence the behaviors of physicians, including their propensity to build collaborative relationships.

The understanding of homophilous relationships is particularly interesting in the healthcare setting, where the final aim is the integration and standardization of healthcare processes. Indeed, the organizational models created to achieve these goals are considered ineffective if they do not encourage interactions among professionals. It is crucial to investigate the determinants of these interactions and to provide the management with an informative basis for evaluating the quality of organizational action. Organizational and social proximity, defined as the closeness among individuals within a certain context or network, make physicians more likely to link closely. Given this proximity, linked physicians become progressively similar because of the induced homophily guiding their choices [24]. For example, Keating et al. [30] showed that physicians are more likely to be influenced by other colleagues of the same organization than by clinicians of other hospitals, suggesting that spatial and geographical proximities do matter for the creation of ties. This influence occurs because belonging to the same social context often means being involved in similar activities or achieving common goals. This trend has also been observed for homophilous ties based on gender. Moreover, prior literature has recorded professional homophilous behaviors between physicians and other categories of healthcare professionals [26], as well as among clinicians of different specialties [5].

Given this theoretical background, in this paper we assume that the social environment in which physicians interact is a relevant factor affecting homophilous ties. More formally, the aim of the present paper is to explore the impact of institutional and professional homophilies on the formation of interphysician professional networks. According to previous literature, people who share similar interests are more likely to form relationships [36-38]. Moreover, sharing a similar context or institutional environment increases mutual trust and the perception of belonging, aspects that influence the formation of interconnections among actors [38,39]. According to this latter definition, the role of the formal organizational structure seems to emerge as a relevant predictor in the creation of interconnections: actors who belong to the same organization are more likely to interact reciprocally, because they have similar problems they need to share and solve. Thus, we define “institutional homophily” the likelihood that individuals will create ties with others who have similar interests and are affiliated with similar institutions.

On the other side, an individual’s professional background could influence interconnections among individuals. In particular, similar perspective and social capital could act as predictors of networks. In this study, we define “professional homophily” as the likelihood that individuals will establish relationships with others who are similar in terms of their field of specialization. We assume that people who generally share a similar background are more likely to create ties.

## Methods

### Research setting

A cross-sectional SNA study was conducted on a community of physicians affiliated with six hospital sites in Bologna’s local health authority (LHA), one of the largest healthcare organizations in the I-NHS. In Italy, LHAs aim to promote and protect the health of all resident citizens in a specific jurisdiction. Currently, there are 145 LHAs representing the basic elements of the I-NHS. Based on the criteria of efficiency and cost-effectiveness, LHAs provide care directly through their own facilities or indirectly by purchasing services from accredited providers, such as independent public and private structures.

The LHA of Bologna serves approximately 800,000 individuals residing in 50 municipalities in the province of Bologna, with more than 80,000 hospitalizations per year. In Bologna’s LHA, hospital activities are carried out according to a matrix organizational model. Six hospital facilities perform hospital activities, which are provided by three clinical directorates. In this LHA, moderately heterogeneous clinical wards and medical specialties are merged into clinical directorates. Moreover, the time elapsed since the new model was formally adopted is satisfactory to ascertain whether behavioral changes, such as new patterns of collaboration between clinicians, are at play.

### Data collection

Data used in this paper refer to a previously published observational study [11,29,40], which was conducted by using a questionnaire survey including 17 questions organized into three main sections^a^. The main purpose of the first section was to collect attributional data on clinicians, including their age, gender, hospital tenure, prior experience in the I-NHS, specialization, and managerial role. The second section was designed to collect data on the information exchange network relationships between clinicians. Consistent with Burt [41], we used an egocentric social network survey instrument to derive a single list of people with whom the respondent had ties. Specifically, each physician was asked to name colleagues both within and outside his/her hospital organization with whom he/she interacts through a) relationships based on exchanging consulting and advice, and b) relationships that are functional for patient assistance. We combined responses into a summary network, including both types of ties. In accordance with a previous study [42], in order to measure the strength of a tie between the respondent and each identified colleague, we asked each respondent “How strong is the connection you have with X?” with possible responses ranging on a 5-point scale from “not at all” (1) to “very much” (5). The purpose of the third section was to determine each clinician’s propensity for adopting EBM. This part of the questionnaire included some questions examining the respondents’ perception of the availability of information and of the possibility of accessing scientific evidence through corporate information technology support.

Questionnaires were submitted online from February to November 2007 to all 329 physicians affiliated with the six hospitals of the LHA. Physicians completed questionnaires during breaks at work or while at home. Participation was voluntary, and respondents were assured that their responses would be confidential and used for research purposes only. According to Italian law, ethics approval was not necessary because no information concerning patients was collected and no experimental research was performed. However, all physicians provided written informed consent for their participation in the study.

### Variables

To achieve the aim of the present study, we used a dyadic approach and assumed the dyad (rather than the individual) as unit of analysis. We used one-mode squared matrices to appraise the relationships among actors in the networks, as well as the differences and similarities among each pair (dyad) of actors [29].

Our dependent variable was relational in nature because it captured interpersonal collaborative relationships between the sampled physicians. Unfortunately, ordinary regression techniques are not suitable to regress the formation of social networks on several independent variables (i.e., interphysician collaborative links). We transformed all covariates representing individual attributes into dyadic explanatory variables. This transformation allowed us to translate attributional data into “relational” data. Specifically, continuous covariates (age, tenure, etc.) were entered into the statistical model as absolute differences between “sender” and “receiver” physician values. Smaller differences indicated greater similarity between physicians, and values of “0” indicated that the physicians were identical with respect to a given attribute. In other words, we assumed that differences in continuous attributes measured the degree of homophily among dyad members, and that positive (negative) signs for continuous variables indicated larger (smaller) differences. Thus, the heterophily (homophily) of physicians positively (negatively) predicted the propensity of physicians to establish collaborative ties with colleagues. In contrast, individual attributes represented by categorical covariates (affiliation with directorates, type of specialization, etc.) or binary covariates (gender, managerial role, etc.) were transformed and entered into the model as binary variables, which took the value “1” if both members of the dyad belonged to the same category, and “0” otherwise.

#### Dependent variable

The interpersonal collaborative network was included in our model by considering a measure of relational “strength” describing the frequency of collaboration among physicians. This dependent variable, named “Professional Network”, was obtained and used to build an adjacency matrix describing the strength of the interconnections among professionals within each dyad with the UCINET 6 software package [43].

#### Explanatory variables

##### Age

Similarity in age occurred when the difference in age between associates was within a range of ±4 years, in accordance with Feld [44]. Age, as a continuous attribute, was measured as the absolute difference in age for each dyad of respondents.

##### Gender

Similarity in gender, as a categorical attribute, was reported as a value of “1” when a pair of actors had the same gender, and a value of “0” otherwise.

##### Seniority

To ascertain whether similarity in seniority was a predictor of professional interconnections, we considered different variables in the model. First, we measured the number of years since graduation, calculating the absolute difference (in years) for each dyad. Second, we measured the tenure within the I-NHS, expressed by the length of service of respondents. Temporal measures were continuous attributes; therefore, we evaluated the absolute difference (in years) for each dyad. Third, we considered the tenure within the LHA that each professional belonged to, measuring the absolute difference (in years) for the dyad. Fourth, we considered whether each professional covered a managerial position (director) or not in his/her organization. Because this attribute was categorical, we assumed the value of “1” when there was similarity in the managerial role in the dyad, and the value of “0” otherwise.

##### Professional homophily

“Professional homophily” refers to the likelihood that a professional will establish relationships with others who are similar in terms of professional interest or who belong to the same field of specialization. Because this attribute was categorical, we reported a value of “1” when similarity in the field of specialization occurred in the dyad, and a value of “0” otherwise.

##### Institutional homophily

“Institutional homophily” refers to the likelihood that a professional will establish relationships with others in the same clinical directorate. This attribute was reported with a value of “1” when both physicians in the dyad were affiliated with the same clinical directorate, and a value of “0” otherwise.

##### Geographical proximity

Physical distance can impact the creation of professional networks and can explain network characteristics [29]. We considered a control variable to explain the physical distance of actors. Data of the respondents’ addresses were obtained, and the absolute value of distances in each dyad were calculated with the Google Maps utility.

### *Estimation technique*

SNA was performed to analyze the collected relational data. SNA is a method of collecting and analyzing data from multiple actors (or nodes) interacting through ties (or edges) with one another [45]. In our case, each physician represented a node, and each edge represented the professional collaboration in service provision.

We performed a multiple regression-quadratic assignment procedure (MR-QAP) to identify predictors of interphysician collaborative ties. MR-QAP is a combinatorial data-analysis procedure adopted routinely in social-network research [9,29,46]. The purpose of MR-QAP is to regress a dependent relational matrix on one or more independent matrices, and to determine whether independent variables are significant predictors of the dependent variables. This procedure is used to model a social relationship matrix by using the values of other relational matrices and control variables, such as attributes of social actors. The MR-QAP procedure was used to ensure that the reported nonparametric estimates would be as robust as possible with respect to our methodological choices. In estimating our models through MR-QAP, we adopted the semi-partially method as reported in Dekker et al. [47], which appears to be reasonably robust against the possible multicollinearity problem that our data may generate. We performed MR-QAP analyses using the UCINET 6 software package [43].

## Results

The sample consisted of 297 physicians (response rate was 90%). All data needed for the analyses were available for the entire sample. Given the relational analytical framework adopted in this paper, the analysis was conducted on 87,912 dyads.^b^ Table 1 shows the correlation coefficients for all variables included in the analyses. Pearson coefficients were computed through quadratic assignment procedures [43].

**Table 1:** Pearson correlation coefficients*

	**Variable**	**1**	**2**	**3**	**4**	**5**	**6**	**7**	**8**	**9**
1	Professional Network	-								
2	Age (difference in)	0.004	-							
3	Gender (same category)	**0.021**	**−0.030**	-						
4	Years since Graduation (difference in)	−0.008	**0.852**	**−0.031**	-					
5	Professional Homophily (same category)	**0.341**	−0.001	**0.035**	0.004	-				
6	Tenure I-NHS (difference in)	0.003	**0.748**	**−0.024**	**0.738**	−0.004	-			
7	Tenure LHA (difference in)	−0.001	**0.427**	−0.001	**0.449**	0.006	**0.532**	-		
8	Managerial Role (same category)	**−0.039**	**−0.171**	**0.024**	**−0.162**	−0.013	**−0.205**	**−0.116**	-	
9	Institutional Homophily (same affiliation)	**0.219**	**0.052**	0.004	−0.014	**0.231**	−0.012	−0.036	0.007	-
10	Geographical Proximity	**−0.066**	−0.010	−0.001	−0.016	**−0.056**	−0.008	−0.008	0.012	**−0.186**

*QAP correlation coefficients (observations = 87,912 - number of permutations = 1,000). Significant coefficients are reported in bold (p < 0.05).

The network variable was slightly correlated with “Gender (same category)” (r = 0.0207, p < 0.05), whereas it was positively correlated with “Professional Homophily (same category)” (r = 0.3414, p < 0.05) and “Institutional Homophily (same category)” (r = 0.2195, p < 0.05). Thus, having the same specialization or belonging to the same clinical directorate increased the likelihood that a link would be observed between two physicians. The network variable was negatively and significantly associated with “Managerial Role (same category)” (r = −0.0395, p < 0.05) and “Geographical Distance” (r = −0.0663, p < 0.05). Thus, physicians who were located further from each other or who had similar roles in their respective organizations were less likely to create collaborative links. A strong positive correlation was found between the variables “Age (difference in)”, “Years since Graduation (difference in),” “Tenure I-NHS (difference in)”, and “Tenure LHA (difference in)”. Therefore, the difference in age between physicians reflected differences in terms of seniority academically, within the I-NHS, and within the currently affiliated organization.

Interestingly, we observed a positive and significant correlation between the two variables capturing the different types of homophily, namely “Professional Homophily (same category)” and “Institutional Homophily (same category)” (r = 0.2315; p < 0.05). Although the level of correlation was moderate, this finding suggest that a certain degree of homogeneity encompassed the criteria for merging clinical wards and hospital specialties into clinical directorates [32].

Table 2 reports the MR-QAP results obtained by regressing all explanatory variables on the professional network. We found that “Professional Homophily (same category)” and “Institutional Homophily (same category)”, representing professional and institutional homophily, respectively, were positively and significantly associated with the dependent variable. The standardized coefficients reported in Table 2 allowed us to compare the diverse impact of the two kinds of homophily. In the model, the coefficient of “Professional Homophily (same category)” (β = 0.1988; p < 0.01) was greater than the coefficient of “Institutional Homophily (same category)” (β = 0.0459; p < 0.01). These results document that professional homophily loomed larger than institutional homophily in predicting the formation of interphysician collaborative network ties in healthcare organizations.

**Table 2:** **MR-QAP estimating factors associated with the propensity of physicians to collaborate**

	**Estimate** ^**†**^	**Significance**
Intercept	0.0000	
Age (difference in)	0.0096	0.242
Gender (same category)	0.0075	0.081
Years since Graduation (difference in)	−0.0318*	0.003
Professional Homophily (same category)	0.1988*	0.000
Tenure I-NHS (difference in)	0.0152	0.126
Tenure LHA (difference in)	0.0030	0.377
Managerial Role (same category)	−0.0307*	0.000
Institutional Homophily (same category)	0.0459*	0.000
Geographical Proximity	−0.0177	0.056
Observations (No. of dyads)	87,912	
Multiple *R* ^*2*^ (Adj.)	0.314 (0.311)	
*p*-value	0.000	

Number of permutations: 5,000; ^†^Standardized coefficients; *p < 0.01.

“Years since Graduation (difference in)” and “Managerial Role (same category)” were significantly associated with the dependent variable of the network. The variable “Years since Graduation (difference in)” was negatively associated with the professional network (β = −0.0318, p < 0.01). Thus, collaborative ties were less likely to be observed between physicians that exhibited larger differences in the time elapsed since graduation. This finding may indicate that attending university during the same time-span can influence the similarity of physicians’ mental models, blueprints, and schemata, which can later affect partner selection within hospital organizations. We believe that this is an important finding deserving further analysis. The negative parameter for “Managerial Role” (β = −0.0307, p < 0.01) indicated that collaborative ties were less likely to be observed between physicians who had the same role in an organization. Clinicians may be requested to integrate and combine different types of knowledge, competences, and responsibilities when establishing collaborative linkages with colleagues. Collaborations between individuals holding different managerial roles may enhance the achievement of a satisfactory level of complementarity in daily hospital activities.

Finally, the coefficient of determination in the model was moderate in degree. This finding clearly indicated that other factors not included in the present study can also explain interphysician collaborative network ties. Prior relationships, friendship ties, and the joint involvement of clinicians in ad hoc organizational task forces or teams are relevant examples of other predictors that future studies should explore.

## Discussion

The flow of clinical knowledge and integration across professional and organizational boundaries is a major challenge for healthcare administrators in many countries. Throughout the world, numerous organizational and technological innovations have progressively dictated hospital restructuring, with the aim of guaranteeing multidisciplinary service provision. New models and practices have been implemented to boost coordination and integration. Notwithstanding their adoption, these interventions risk remaining largely ineffective if they do not impact on how physicians communicate and interact [1]. This study aimed to disentangle the formation process of interactions by examining the professional network in a community of physicians working in an Italian LHA. Homophily theory and SNA provided the theoretical and instrumental techniques for identifying theory, measures, and empirical models.

We tested how institutional homophily (belonging to the same clinical directorate) and professional homophily (belonging to the same medical specialty) affect the propensity of physicians to establish a connection. As documented, homophily can arise from the social context (“induced homophily”) or from individual preference (“choice homophily”) [21]. In our perspective, institutional homophily clearly correlates with the reference social context, which the management contributed to (re)shape by introducing a new organizational model. Conversely, professional homophily represents a mixed composition of the two perspectives, as the choice of the medical field is originally due to an individual choice.

Our first result is that homophily matters in the formation of connections within healthcare organizations. This finding is consistent with numerous studies in other settings, which have found that homophily strongly affects connections. People expect, a priori, that self-similar colleagues are more likely to accept them, be trustworthy, and hold similar beliefs, thereby mitigating the potential conflicts, misunderstandings, and monitoring costs that come with making connections [19,36].

Our second result is that the homophily effect related to specialty (professional homophily) is stronger than the effect related to organizational structure (institutional homophily). This finding is new and may be explained by the theoretical arguments of professionalism in healthcare. The culture, beliefs, wisdom, and cognitive mental models of physicians are formed well before they enter healthcare organizations [48]. As documented by Ferlie et al. [5], complex organizations contain many different professional groups, each of which may operate in a distinct community of practice. Categories of professionals are typically separated from each other by social and cognitive boundaries, which may be an impediment to the creation of trustworthy relationships. Medical specialties represent the first example of professional categories which physicians may belong to. These specialties largely contribute to explain physicians’ propensity to establish advice networks and peer-to-peer communication in hospital organizations [30]. In this setting, boundaries delineate differences between categories in terms of professional norms, which can delay or prevent knowledge sharing and diffusion among professionals belonging to different knowledge domains [5].

The current study has some limitations. First, network data were collected and analyzed at one point in time. As observed, network configurations may be influenced by previous patterns of relationships. Thus, future longitudinal studies are needed to extend the validity of our results. Second, we demonstrated that people are more likely to create social ties with self-similar others; however, no research has investigated whether, over time, people are more likely to maintain homophilous or heterophilous relationships. Third, random sampling was not used. However, because of the large organizational context of the study, the characteristics of the investigated organization, and the quality of the collected data in terms of compliance, we believe that our results might be extended to analogous organizations within the I-NHS. Yet, the generalizability of this piece of evidence to other healthcare contexts remains limited.

Fourth, institutional homophily is likely influenced by the timing of adoption of new arrangements and models. Professionals are uncertain about the costs and benefits of a new model at the time of its adoption. As time elapses, clinicians will be more likely to acknowledge and understand organizational innovations. Moreover, the process of identifying physicians as relevant partners may change over time. Homophily is highly dynamic, subject to change, and influenced by exogenous forces that affect the processes of individuals’ social identification [49]. The adoption of new clinical directorates delineates new organizational boundaries, which will likely affect physicians’ perceptions about who are heterophilous colleagues and their mental predisposition towards homophilous colleagues. The impact of such internal redesign on physicians’ identification and perception of homophily may eventually overshadow their traditional perception about homophilous colleagues (i.e., individuals holding the same clinical specialty). Future studies are encouraged to explore whether professional and institutional homophilies have different impacts on partner selection by physicians, by comparing two or more organizations in which clinical directorates are adopted at different times. Finally, to test professional homophily, we only included actors within the organization under study, and excluded potential links to physicians outside our setting. Further research should include actors outside the organization, to see whether the results are similar to our own.

## Conclusions

This study proposes and applies homophily theory as a way to capture the formation of professional and institutional networks among physicians. SNA revealed the interpersonal communication structure, which could not be visualized by conventional surveys.

Our findings have several implications for hospital executives. Managing intraorganizational networks involves two opposite management tasks. Managerial interventions are required because organizations primarily use these networks to integrate dispersed knowledge. On the other hand, the networks are strongly self-organizing and emergent in nature, independent from (or even negatively influenced by) interventions by management [50]. To be effective, management strategies aimed at increasing networking among employees should not focus solely on the creation of organizational models, as has often been done, but also should focus on the creation of a need to access others’ resources [9] (clinical guidelines, clinical audits, etc.). Second, managers are encouraged to foster collaboration across heterogeneous groups of physicians characterized by different specializations: for example, by targeting and defining group objectives, adopting new organizational arrangements, or restructuring processes [40]. Another example in this direction is the adoption of specific types of clinical directorates or interdisciplinary and interprofessional groups [35]. Clinical directorates could organize training programs that encourage multidisciplinarity, as well as informal occasions of socialization addressed to foster collaborative relationships among physicians.

## Endnotes

^a^ The questionnaire is available from the authors upon request.

^b^Relational data are generically represented by squared “n × n” sociomatrices, where “n” is the number of actors. Our 297 sampled clinicians eventually generated a sample of 87,912 dyads, which equals “297 × 297” (excluding the main diagonal of the matrix).

## Abbreviations

EBM: Evidence-based medicine
I-NHS: Italian National Health Service
LHA: Local health authority
MR-QAP: Multiple regression quadratic assignment procedure
SNA: Social network analysis
